# Commentary: Dengue fever: a decade of burden in Iran

**DOI:** 10.3389/fpubh.2025.1553489

**Published:** 2025-05-09

**Authors:** Mohammad Mehdi Sedaghat

**Affiliations:** Department of Vector Biology and Control of Diseases, School of Public Health, Tehran University of Medical Sciences, Tehran, Iran

**Keywords:** dengue fever, *Aedes (Ae) aegypti*, *Aedes albopictus*, vector control, health sanitation

In this paper we critically review the work “*Dengue fever: a decade of burden in Iran*” by Heydarifard et al. (1) published in Frontiers in Public Health (12:1484594- 2024). This was a very interesting article and I would like to congratulate the authors on their work while also contributing a few comments.

The article provides data from epidemiological studies of the dengue virus (DENV) in Iran, tracing its spread from the first reported case in a traveler until July 2024. The authors state that DENV “has gradually spread across Iran” (1). While this statement is accurate for the past, the situation has become more complex, as Iran is now experiencing sudden outbreaks originating from endemic areas in the region. It is important to note that the first two locally acquired cases were reported in June 2024 (2). In recent years, the primary vectors of dengue fever, *Aedes aegypti* and *Ae. albopictus*, have entered Iran, adding to this complexity. The reappearance of *Ae. aegypti* was confirmed in Hormozgan Province in 2018 (3), and its distribution has since gradually extended through southern Iran. Similarly, *Ae. albopictus* has begun to expand its presence in northern parts of the country (4). While the authors have mentioned the possibility of *Ae. albopictus* establishing itself in the southeast corner of the country, it is important to highlight that there have been no documented reports of its establishment or distribution in that area since its initial observation (3–5). The presence of *Ae. albopictus* in northern Iran is a topic of growing importance, and it is essential to rely on the most current and relevant information available. While some may reference older documents, recent data and insights from Iran CDC (ICDC) and the Ministry of Health and Medical Education, along with contributions from independent scientists, clearly indicate that this species is now well-established in the northern parts of Iran (3, 4, 6, 7). While the review article does not mention this fact, it is crucial for health authorities, scientists, and the general public to be aware of its occurrence and expansion. Understanding these aspects is essential for assessing their implications for public health and vector management.

The review article highlights various studies conducted in Iran and presents some recommendations regarding vector control and health education. The article does not address several critical components related to these subjects, particularly an important control method, health sanitation. This oversight limits the reader's ability to fully understand the situation and identify effective prevention and control strategies. After reading the article, one would expect to gain a clear perspective on the current transmission and circulation of DENV between humans and vectors. According to the ICDC reports and our extensive field visits, along with monitoring the surveillance system in Iran, there are confirmed local transmissions of dengue fever in two regions: Chabahar and Port Lengeh. The risk of transmission is currently high only in these areas. In regions of the country where the *Ae. aegypti* or *Ae. albopictus* are present, if a patient from an endemic area is imported, the *Aedes* mosquitoes can become infected and enable the spread of dengue virus (DENV) (2–4, 7). In most parts of the country where these mosquito vectors are not reported, there is no risk of transmission (2–4). However, given the presence of these two significant vectors, it is expected that Iran will soon experience an increase in dengue cases, leading to potential epidemics and outbreaks.
